# Late effects in survivors of childhood acute lymphoblastic leukemia in the context of selected gene polymorphisms

**DOI:** 10.1186/s13052-018-0526-5

**Published:** 2018-08-15

**Authors:** Kinga Kwiecinska, Wojciech Strojny, Danuta Pietrys, Miroslaw Bik-Multanowski, Maciej Siedlar, Walentyna Balwierz, Szymon Skoczen

**Affiliations:** 1 Department of Oncology and Hematology, Institute of Pediatrics, Jagiellonian University Medical College, Wielicka 265, 30-663 Krakow, Poland; 2 Department of Medical Genetics, Institute of Pediatrics, Jagiellonian University Medical College, Krakow, Poland; 3 Department of Clinical Immunology, Chair of Clinical Immunology and Transplantation, Institute of Pediatrics, Jagiellonian University Medical College, Krakow, Poland

**Keywords:** Late effects, Acute lymphoblastic leukemia, Gene polymorphisms

## Abstract

**Background:**

It has been shown that approximately half of survivors of childhood acute lymphoblastic leukemia (ALL) have symptomatic late effects (LE) that may be severe or life-threatening. The aim of our study was to assess the health status of childhood ALL survivors after over 10 years of follow-up and to assess its relationships with gene polymorphisms, numbers and types of LEs, as well as with intensity of chemotherapy and cranial radiotherapy (CRT).

**Methods:**

We conducted a telephone survey in 125 ALL survivors (median time from completion of treatment was 12 years) and compared the results with those obtained in our previous study. Most of the patients were followed-up by local providers.

**Results:**

The prevalence of LEs of approximately 50% was similar in both study groups. More than one LE was found in almost 25% of patients. Endocrine LEs were less frequent than in our previous study (44% vs 22%), probably due to underdiagnosis. The prevalence of hepatitis B/C decreased from 30%/50 to 18% (counted together), and prevalence of neurologic LEs decreased from 18 to 6%. The increase in the rate of second malignancies was not significant (2% vs. 3%). Sixty four percent of patients continued their education at the time of the study. Approximately 51% of ALL survivors who have completed their education by the time of the study had no permanent employment, including 4 mothers of infants and 3 persons qualified for a disability living allowance. These employment problems may have been due to cognitive impairment. The offspring of the ALL survivors included 11 children, all of them healthy. Further analysis showed higher prevalence of hepatitis in patients treated with CRT (*p* = 0.0001). Genetic studies revealed higher prevalence of hepatitis in patients homozygous for the rs9939609A variant of the *FTO* gene compared with other patients (*p* = 0.03). Moreover, wild-type rs1137101 polymorphism (Q223R) of the and leptin receptor gene was more frequent in patients with psychological LEs (*p* = 0.03).

**Conclusions:**

The prevalence of LEs in ALL survivors is of key importance. The transition of childhood ALL survivors from pediatric to adult care should be urgently improved to maintain continued follow-up provide high-quality care.

**Trial registration:**

Bioethics Committee of the Jagiellonian University approved the study protocol. Registration number: KBET/113/B/2006.

## Background

In our previous study [1], conducted from 1994 to 2000 in the Late Effects Outpatient Clinic of the Department of Oncology and Hematology, Institute of Pediatrics, Jagiellonian University, Krakow, Poland, we studied 255 patients, aged 4 to 28 years (median 12 years), who have completed acute lymphoblastic leukemia (ALL) treatment. The median time from completion of the treatment to enrollment in the study was 3 years. More than 50% of ALL survivors had late effects (LE) of treatment. The most frequent LEs were cognitive impairment and abnormalities of growth and body weight. Second neoplasms were diagnosed in 2% of patients. Endocrine LEs of any type were observed in 40% of ALL survivors. Other LEs were thyroid disorders, hypogonadism, premature puberty, seen in 11, 3 and 1% of patients, respectively. Menstruation disturbances were observed in 2% of female patients. Results of psychological tests of the ALL survivors were below the level of their healthy peers, mainly in mathematical skills, and the rate of test processing was slower. Girls had worse results than boys, and cranial radiotherapy (CRT) was implicated as the main cause of the impairments. Neurological LEs were seen in 18% of patients, and behavioral problems in 20% of patients. Serological markers of hepatitis B virus infection were positive in more than 30% of patients, and markers of hepatitis C virus infection in almost 50% of patients. Patients were followed-up in the clinics of the Institute of Pediatrics until they were 18 years old, and then they were referred to an adult outpatient center at the University Hospital in Krakow. The aim of our study was to assess the health status of childhood ALL survivors after more than 10 years of follow-up.

Polymorphisms of the genes responsible for regulation of metabolism can be associated with various LEs [2–4]. As the regulation of the insulin pathway is responsible for proliferation of cell lineages [5, 6], the role of polymorphisms of the genes involved in this pathway in the emergence of LEs of ALL treatment is interesting. In vitro, recombinant FTO takes part in catalyzing demethylation of certain methyl nucleotides in single-stranded DNA and RNA, which suggests a potential role of FTO in nucleic acid repair or modification. The *FTO* gene polymorphism is associated with an increase in the risk of obesity, as well as type 2 diabetes, heart failure, coronary heart disease, lifetime all-cause and ischemic stroke, hypertension, dyslipidemia, metabolic syndrome, and mortality. Moreover, it is also associated with increased fasting glucose and insulin levels, 2-h OGTT test results, HbA1c, blood pressure, lipid levels, liver function tests, and inflammatory markers, which are potential risk factors of the disorders mentioned above [7]. Obesity is also considered a risk factor for certain cancers [8]. Leptin receptor gene (*LEPR*) encodes the leptin receptor that is activated by leptin and has effects on food intake in humans. Mutations of the leptin gene and leptin receptor gene in obese patients suggest that leptin takes part in regulation of energy balance [9]. *LEPR* gene polymorphisms, K109R and Q223R, located in exons 4 and 6, are implicated in the regulation of lipid metabolism and insulin resistance [10]. Genetic associations between these two polymorphisms and obesity parameters, including insulin resistance, glucose levels and serum lipid profile, have been reported [11, 12]. Moreover, Q223R polymorphism of the *LEPR* gene is associated with metabolic syndrome [13].

Based on this theoretical background we investigated associations between the selected gene polymorphisms and prevalence of LEs in pediatric ALL survivors.

## Methods

The study cohort included 125 patients who have previously completed ALL treatment and were available for a telephone survey conducted by a physician working at the Outpatient Clinic of the Department of Oncology and Hematology, Institute of Pediatrics, Jagiellonian University. Majority of them (65 out of 125) were included in our previous study. ALL therapy was conducted from 16/01/1984 to 8/11/2004 according to a modified BFM regimen with subsequent revisions (100 patients) or a New York regimen (25 patients). Details concerning treatment regimens were published elsewhere [14–16]. Age at ALL diagnosis was 1–18.5 years (median 4.4, mean 4.2 years.) Details of the patients are provided in Table 1.

**Table 1:** Characteristics of the study group

Parameter	All patients	Cranial radiotherapy	No cranial radiotherapy
Total (*n*)	125	61	64
Female	67	33	34
Male	58	28	30
Treatment regimen (*n*)			
BFM	100	37	63
New York	25	24	1
Relapses	5	4	1
CNS	3	2	1
Testes	2	2	0
Intensity of treatment			
regimen (n)			
High intensity	30	30	0
Low intensity	95	28	67
Age at ALL diagnosis (years)	1–18.5 (median 4.4)	1.9–18.5 (median 4)	1–10.3 (median 4.8)
Age at enrollment (years)	10.6–33 (median 20.3)	12.2–33 (median 24.7)	10.6–25.6 (median 17.7)
Time from completion of ALL treatment (years)	4.3–25.7 (median 11.7)	4.3–25.7 (median 15)	5.1–21 (median 10.3)

All patients included in the survey were interviewed by the same physician and the questions concerned: education/employment, health problems, established diagnoses, recent outpatient visits or hospital admissions, medications in use, marital status and offspring.

Data regarding the polymorphisms studied in the patients were previously published elsewhere [17, 18].

Correlations between gene polymorphisms and the numbers and types of LEs were analyzed with respect to the intensity of chemotherapy (less intensive BFM regimens for standard/ intermediate-risk patients vs. more intensive New York regimens for high-risk patients, and BFM regimen for relapsed ALL) and to CRT. Descriptive statistics, odds ratios (OR) with 95% confidence interval, and Fisher’s exact test were used.

Local bioethics committee approved the study protocol. All parents and adolescent patients signed an informed consent before blood sample collection.

## Results

The study cohort included 125 survivors of childhood ALL aged from 10.6 to 33 years (median 20.3 years); 58 patients (46%) were male and 67 patients (54%) were female. In 61 patients CRT was administered (doses: 14 to 24 Gy; median 18.2 Gy) according to the treatment regimens. A second CRT was performed in 3 patients (15 Gy, 18 Gy and 18.2 Gy), and spinal radiotherapy in one patient. Testicular radiotherapy was used in 2 patients (18 Gy and 21 Gy). In 95 (76%) patients less intensive ALL treatment regimens were used, and 30 (24%) patients were treated according to the more intensive regimens. Two patients completed a second course of treatment for relapsed ALL. One of them was treated with hematopoietic cell transplantation from a matched sibling donor. The duration of ALL treatment was from 1.7 to 4.2 years (median 3.2 years).

LEs were seen in 65 patients (52%), including one LE in 35 patients (28%), and > 1 LE in 30 patients (24%). Two LEs were seen in 20 patients (16%), 3 LEs in 8 patients (6%), and 5 and 7 LEs in 1 patient each (about 1%).

The most frequent LEs (Table 2) were endocrine disturbances, hepatitis, psychological and neurological abnormalities, which were seen in 22, 18, 10 and 6% patients, respectively. Further analysis revealed increase in the prevalence of hepatitis in patients treated with CRT (*p* = 0.0001).

**Table 2:** Late effects of ALL treatment

Late effect	*n*	%
Endocrine – hyperthyroidism, hypothyroidism, hyperprolactinemia, testosterone deficiency, growth hormone deficiency, obesity, infertility	27	22
Hepatitis B or C	23	18
Psychological – dyslexia, impairment of cognitive function and memory	12	10
Neurological – recurrent headache, epilepsy	7	6
Gynecological – menstruation disorders	6	5
Psychiatric - depression, phobia, anorexia	4	3
Second neoplasm – meningioma (2 cases), cervical cancer, haeeamangioma of the liver	4	3
Musculoskeletal – bone and muscle pain, vertebral column pain, scoliosis, osteoporosis	9	7
Allergy	3	2
Dermatological – psoriasis, eczema	3	2
Nephrological – nephrolithiasis, nephropathy	3	2
Gastrointestinallogical – cholelithiasis, gastritis	3	2
Pulmonary – asthma, recurrent pneumonia	2	1.6
Ocular – diplopia	1	0.8
Cardiovascular – hypertension, hypotension	2	1,6
Ear, nose throat – hearing impairment	1	0.8
Immunological - immunodeficiency	1	0.8

Sixty four percent of the patients continued their education. About 51% of the patients who have completed their education had no permanent employment, including 4 mothers of children below 1 year of age and 3 persons who have qualified for a disability living allowance (Table 3).

**Table 3:** Employment/education status in ALL survivors

Status	*n*	Additional information
Disability pension	3	
Unemployment	20	Education level: Primary school – 11 Trained to profession after primary school – 3 Technical primary school – 3 Technical high school – 1 High school – 1 University – 1
Physical labor	2	Primary school
Employment after completing education	4	Technical primary school
	8	High school/Technical high school
	8	University
Primary school	12	Education in progress
Primary technical school	3	
Secondary school	23	
High school/Technical high school	22	
University	20	

The offspring of ALL survivors included 11 children (age from 0.3 years to 6 years; 7 girls and 4 boys). All of them were healthy.

Genetic studies revealed higher prevalence of hepatitis in individuals homozygous for the rs9939609A variant of the *FTO* gene compared with other patients (*p* = 0.03). Moreover, psychosocial complications were seen more frequently in patients with wild-type rs1137101 polymorphism (Q223R) of *LEPR* gene (*p* = 0.03). Numbers of patients in whom the studied polymorphisms were found are presented in Tables 4 and 5.

**Table 4:** Rates of the studied polymorphisms

	Genotype		
FTO gene polymorphism rs9939609 (c.28-23,525 T > A)	TT	TA	AA
Total (n)	58	36	31
CRT+	25	17	17
CRT-	33	19	14
Leptin receptor gene (LEPR) polymorphism rs1137100 (K109R; c.326A > G)	AA	AG	GG
Total (n)	58	59	8
CRT+	30	27	4
CRT-	28	32	4
Leptin receptor gene (LEPR) polymorphism rs1137101 (Q223R; c.668A > G)	AA	AG	GG
Total (n)	69	28	28
CRT+	30	16	15
CRT-	39	12	13
Leptin receptor gene (LEPR) polymorphism rs1805094 (K656 N; c.1968G > C)	GG	GC	CC
Total (n)	25	99	1
CRT+	10	50	1
CRT-	15	49	0

*CRT* cranial radiotherapy

**Table 5:** Genotypes of the FTO and LEPR variants in patients with the most common LEs; statistical analysis using Fisher’s exact test (NS – not significant)

FTO gene polymorphism rs9939609 (c.28-23,525 T > A)				
LEs	Genotypes Number of cases with LEs / without LEs			Statistical significance (Fisher’s exact test)
	TT	TA	AA	
Endocrine disturbances	7/29	11/47	7/24	NS
Hepatitis	9/27	4/54	10/21	*p* *= 0.004 (AA vs. TT);* *p* *= 0.03 (AA vs. AT + TT)*
Psychological abnormalities	2/34	8/50	1/30	NS
Leptin receptor gene (LEPR) polymorphism rs1137100 (K109R; c.326A > G)				
LEs	Genotypes Number of cases with LEs / without LEs			Statistical significance (Fisher’s exact test)
	AA	AG	GG	
Endocrine disturbances	9/49	14/45	2/6	NS
Hepatitis	12/46	9/50	2/6	NS
Psychological abnormalities	8/50	4/55	0/8	NS
Leptin receptor gene (LEPR) polymorphism rs1137101 (Q223R; c.668A > G)				
LEs	Genotypes Number of cases with LEs / without LEs			Statistical significance (Fisher’s exact test)
	AA	AG	GG	
Endocrine disturbances	16/53	5/23	4/24	NS
Hepatitis	11/58	6/22	6/22	NS
Psychological abnormalities	11/58	1/27	0/28	*p* *= 0.03 (AA vs. GG)*
Leptin receptor gene (LEPR) polymorphism rs1805094 (K656 N; c.1968G > C)				
LEs	Genotypes Number of cases with LEs / without LEs			Statistical significance (Fisher’s exact test)
	GG	GC	CC	
Endocrine disturbances	6/19	19/80	0/1	NS
Hepatitis	3/22	19/80	1/0	NS
Psychological abnormalities	2/23	10/89	0/1	NS

Statistically significant differences were found in the prevalence of hepatitis in patients with FTO gene polymorphism rs 9939609 (c.28- 23,525 T>A) – *p* = 0.004 (AA vs. TT); *p* = 0.03 (AA vs. AT + TT) and in the prevalence of psychological abnormalities in patients with LEPR polymorphism rs1137101 (Q223R; c.668A > G) – *p* = 0.03 (AA vs. GG)

In the analysis of the entire study cohort, no statistically significant differences in the studied gene variants and ALL treatment intensity were found.

## Discussion

Currently, a combined multimodal therapy (multi-agent intensive chemotherapy, radiotherapy and stem cell transplantation) allows for achieving a complete cure in approximately 90% of children with ALL [19]. Growing numbers of young and middle-aged (15–45 years) childhood cancer survivors require skilled professionals to take care of this category of patients. In 1990, one in 1000 young adults was a survivor of childhood cancer, whereas in 2010 the rate was one in 250 [20]. In 1994 Childhood Cancer Survivor Study (CCSS) was started in the USA. It is still ongoing and it currently includes follow-up data of more than 20,000 childhood cancer survivors who have completed therapy more than 5 years ago [21]. It was shown that approximately half of the survivors have symptomatic LEs, including severe and life threatening LEs in 21 and 14% of them, respectively [22]. The LEs include: premature mortality, a second malignant neoplasm, organ dysfunction (e.g. heart, lung, gonads), growth impairment, delayed puberty, infertility, impairment of cognitive function. These may cause employment and insurance problems and impaired quality of life [20, 22–46].

Use of radiotherapy and the type of cancer are the key risk factors for LE development [20, 22, 23]. Prevalence of LEs was higher in patients treated for solid tumors compared with those treated for leukemia or lymphoma [20, 22].

It is estimated that 1 in 715 young adults in the UK are survivors of childhood cancer and ALL was the diagnosis in 15% of the survivors aged from 20 to 39 years [47]. About 28% of ALL survivors have psychological sequelae [48]. Chronic conditions are found in 66 to 88% of childhood cancer survivors, and their prevalence increases with age [43]. The prevalence of LEs in our cohort was within the ranges reported in the literature. However, given the methodology of our study, we may have underestimated the prevalence of LEs in our study population.

In the present study we assessed the prevalence of LEs using a telephone survey conducted in patients who have previously been treated at our center. This is an important limitation of the study. However, the calls were performed by the same physician who has previously taken care of the patients at the Late Effects Outpatient Clinic, which in turn improves the quality of the study. We were able to reach approximately 50% of the patients included in our previous study in the late 1990s. The difference in a median follow-up between both studies was approximately 9 years (3 vs 12 years). Only a few patients remained in a continued care of an outpatient clinic for adult survivors of childhood cancer. Almost all other patients were in a care of local healthcare providers. Prevalence of LEs in both studies was the same (50%). More than one LE was found in almost 25% of the patients. Endocrine LEs were less frequently seen in our present study (44% vs 22%). This was probably due to underdiagnosis. The prevalence of hepatitis B/hepatitis C decreased from 30%/50 to 18% (counted together), and prevalence of neurological LEs decreased from 18 to 6%. The increase in the rates of second malignant neoplasms was not significant (2% vs. 3%). The study revealed higher prevalence of hepatitis in patients treated with CRT (*p* = 0.0001) and in patients homozygous for the rs9939609A variant of the *FTO* gene (which in our previous study was associated with higher BMI) compared with other patients. This may be explained by the use of CRT in the more intensive treatment regimens, which increased the risk of infection, whereas obesity, which was more prevalent in patients homozygous for rs9939609A variant of the *FTO* gene, causes impaired liver function due to activation of the insulin pathway and may be associated with higher susceptibility to infections. However, we are unable to explain why wild-type Q223R polymorphism of *LEPR* gene was more frequently found in patients with psychological LEs (*p* = 0.03). Interestingly, as in our previous study, we were unable to establish correlations between the tested polymorphisms and the development of LEs in the context of intensity of chemotherapy. It was probably due to a limited number of patients included in the study. In the future, genetic studies (assessment of gene expression profile, GWAS) might be useful in stratification of patients, personalization of therapy, outcome prediction and estimation of the risk of LEs, as well as in identification of new therapeutic targets.

In our previous study, quality of life was assessed using a score (1–5) defined by Skoczen [1]. The average score was 4.4 points. Unfortunately, the score was not included in the current telephone survey questionnaire.

Sixty four percent of childhood ALL survivors continued their education. Approximately 51% of patients who completed the education had no permanent employment, including 4 mothers of children below 1 year of age and 3 persons who have qualified for a disability living allowance. The offspring of the ALL survivors included 11 children (age from 0.3 years to 6 years; 7 girls and 4 boys). All of them were healthy.

As it was shown in other studies, cognitive impairment may be the reason for employment problems [23, 30, 35]. The unemployment rate in the study cohort was significantly higher than in age-matched general population in Poland (11% unemployed, 15% temporarily unemployed) [49]. The levels of education of survivors of childhood malignancies are generally lower than in the healthy population of the same age. In our study, a university degree was achieved by 20% of patients who completed their education, compared with 31% in the age-matched Polish population [50]. One of the most important issues in many pediatric oncology centers is the transition of patients > 18 years of age to adult oncology centers [51, 52]. Our procedure is to refer the survivors to the University Outpatient Clinic. Unfortunately, most of our patients are not satisfied with the care offered by the adult clinic. Therefore, they are most frequently followed-up by local healthcare providers, who have no skills necessary for comprehensive care of childhood cancer survivors. This may cause frequent underdiagnosis of LEs due to the limited awareness. This is the first study of survivors of childhood ALL that describes the current situation of this group of patients in Poland. We found shortages of appropriate medical care of these patients due to a lack of awareness in patients, physicians and health care providers. Because of inefficient organization and limited resources, the diagnosis and treatment of LEs were delayed.

Well-designed protocols regulating the transition of patients from pediatric to adult outpatient care are necessary to improve this situation. Outpatient clinics should include multidisciplinary care covering most common LEs. Appropriate communication and data exchange between pediatric and adult healthcare providers should be ensured, with joint discussion on specific recommendations in particular patients, where necessary. It would be beneficial for the childhood cancer survivors, particularly those who have completed their therapy long time ago, to participate in conferences and use internet resources to provide them with access to comprehensive information on their health, quality of life, education and employment.

## Conclusions

The prevalence of LEs in childhood ALL survivors remains a crucial issue. Most of the patients are inadequately followed-up. The transition of pediatric patients to adult care should be urgently improved to maintain long-term follow up and provide high quality of care. Higher prevalence of hepatitis in individuals homozygous for the rs9939609A variant of the *FTO* gene might be associated with prolonged impairment of liver function thus causing higher susceptibility to infections.

## Abbreviations

ALL: Acute lymphoblastic leukemia
BFM: Berlin-Frankfurt-Münster
CCSS: Childhood Cancer Survivor Study
CNS: Central nervous system
CRT: Cranial radiotherapy
FTO: Fat mass and obesity-associated protein
LE: Late effects
OGTT: Oral glucose tolerance test
OR: Odds ratio
